# County-Level Food Insecurity and Access to Medicare Advantage Food Benefits

**DOI:** 10.1001/jamanetworkopen.2025.48223

**Published:** 2025-12-10

**Authors:** Manish Kumar, Amelia M. Bond, Dhruv Khullar, William L. Schpero

**Affiliations:** 1 Division of Health Policy and Economics, Department of Population Health Sciences, Weill Cornell Medical College, New York, New York; 2 Cornell Health Policy Center, Cornell University, New York, New York; 3 Division of General Internal Medicine, Department of Medicine, Weill Cornell Medical College, New York, New York; 4 The Physicians Foundation Center for the Study of Physician Practice and Leadership, Weill Cornell Medical College, New York, New York; 5 Cornell Center for Health Equity, Cornell University, New York, New York

## Abstract

This cross-sectional study examines whether there is an association between county-level rates of food insecurity and the availability of food benefits through Medicare Advantage plans.

## Introduction

Nearly 17% of Medicare enrollees experience food insecurity,^1^ a share projected to increase following recent cuts to the Supplemental Nutrition Assistance Program.^2^ Since 2020, Medicare Advantage (MA) plans—which now enroll more than half of the Medicare population—have been able to offer a Food and Produce supplemental benefit, allowing plans to provide targeted allowances to beneficiaries to purchase healthy foods.

Supplemental benefits have drawn scrutiny, given mixed and incomplete evidence on how they are used and to whom they are offered.^3,4,5^ We examined the characteristics of plans offering the Food and Produce benefit in 2025 and assessed whether enrollment in these plans matched county-level need for nutritional support.

## Methods

For this cross-sectional study, data on county-level MA plan characteristics, including enrollment and Food and Produce benefit offerings, came from the Centers for Medicare & Medicaid Services (CMS) 2025 MA public use files. County-level estimates on the proportion of residents who were food insecure came from Feeding America’s Map the Meal Gap.^6^

We used 2-sample tests of proportion to assess for differences in the characteristics of plans offering the Food and Produce benefit relative to those not offering the benefit. We examined the county-level association between food insecurity and the proportion of MA beneficiaries enrolled in plans offering the Food and Produce benefit using ordinary least squares regression, sequentially adjusting for county-level plan characteristics to isolate whether plan availability or enrollment drove any observed associations. We defined significance as *P* < .05 with Bonferroni corrections for multiple comparisons.

This study was deemed not human participant research by the institutional review board at Weill Cornell Medicine and followed STROBE reporting guidelines for cross-sectional studies. Analyses were performed using Stata version 18.5 (StataCorp). See eMethods in Supplement 1 for additional details.

## Results

The sample included 5105 MA plans across 3064 counties (median [IQR] food insecurity, 15.1% [12.8%-17.6%]); 33.8% of plans offered the Food and Produce supplemental benefit (Table). Plans that offered Food and Produce benefits, relative to those that did not, were more likely to be health maintenance organizations (difference, 22.8 percentage points [PP], 95% CI, 19.9-25.6 PP; *P* < .001), newer (contract effective date >2020: difference, 9.8 PP, 95% CI, 7.3-12.3 PP; *P* < .001), smaller (lowest quartile enrollment: difference, 7.6 PP; 95% CI, 5.0-10.2 PP; *P* < .001), and Special Needs Plans (SNPs; difference, 62.2 PP, 95% CI, 59.8-64.5 PP, *P* < .001). Among plans offering the benefit, 82.7% did not require prior authorization to access the benefit.

**Table.** Characteristics of Medicare Advantage Plans Stratified by Offering of Food and Produce Supplemental Benefits, 2025

Characteristic	Plans, No. (%)^a^			Difference (95% CI), percentage points	*P* value
	With Food and Produce benefit	No Food and Produce benefit	Total		
Total	1727 (33.8)	3378 (66.2)	5105 (100.0)	NA	NA
Plan type					
HMO	949 (55.0)	1087 (32.2)	2036 (39.9)	22.8 (19.9 to 25.6)	<.001
HMO-POS	404 (23.4)	762 (22.6)	1166 (22.8)	0.8 (−1.6 to 3.3)	.50
PFFS	1 (0.1)	20 (0.6)	21 (0.4)	−0.5 (−0.8 to −0.3)	.005
Local PPO	370 (21.4)	1461 (43.3)	1831 (35.9)	−21.8 (−24.4 to −19.3)	<.001
Regional PPO	3 (0.2)	48 (1.4)	51 (1.0)	−1.2 (−1.7 to −0.8)	<.001
Plan parent organization					
Humana Inc	214 (12.4)	511 (15.1)	725 (14.2)	−2.7 (−4.7 to −0.8)	.008
UnitedHealth Group Inc	266 (15.4)	609 (18.0)	875 (17.1)	−2.6 (−4.8 to −0.5)	.02
CVS Health Corporation	267 (15.5)	428 (12.7)	695 (13.6)	2.8 (0.7 to 4.8)	.006
Centene Corporation	93 (5.4)	226 (6.7)	319 (6.2)	−1.3 (−2.7 to 0.1)	.07
Elevance Health Inc	186 (10.8)	150 (4.4)	336 (6.6)	6.3 (4.7 to 7.9)	<.001
Other	701 (40.6)	1454 (43.0)	2155 (42.2)	−2.5 (−5.3 to 0.4)	.09
Overall Star rating					
<3.5	223 (12.9)	480 (14.2)	703 (13.8)	−1.3 (−3.3 to 0.7)	.20
3.5 to 4.0	938 (54.3)	1895 (56.1)	2833 (55.5)	−1.8 (−4.7 to 1.1)	.23
4.5	306 (17.7)	732 (21.7)	1038 (20.3)	−4.0 (−6.2 to −1.7)	.001
5.0	20 (1.2)	67 (2.0)	87 (1.7)	−0.8 (−1.5 to −0.1)	.03
Unavailable	240 (13.9)	204 (6.0)	444 (8.7)	7.9 (6.0 to 9.7)	<.001
Contract effective date					
<2006	621 (36.0)	1573 (46.6)	2194 (43.0)	−10.6 (−13.4 to −7.8)	<.001
2006 to 2013	370 (21.4)	748 (22.1)	1118 (21.9)	−0.7 (−3.1 to 1.7)	.56
2014 to 2019	247 (14.3)	433 (12.8)	680 (13.3)	1.5 (−0.5 to 3.5)	.14
>2020	489 (28.3)	624 (18.5)	1113 (21.8)	9.8 (7.3 to 12.3)	<.001
Enrollment^b^					
<576 Individuals	519 (30.1)	759 (22.5)	1278 (25.0)	7.6 (5.0 to 10.2)	<.001
576-1800 Individuals	402 (23.3)	874 (25.9)	1276 (25.0)	−2.6 (−5.1 to −0.1)	.04
1801-5586 Individuals	382 (22.1)	893 (26.4)	1275 (25.0)	−4.3 (−6.8 to −1.9)	.001
>5586 Individuals	424 (24.6)	852 (25.2)	1276 (25.0)	−0.7 (−3.2 to 1.8)	.60
SNP					
Yes	1191 (69.0)	229 (6.8)	1420 (27.8)	62.2 (59.8 to 64.5)	<.001
No	536 (31.0)	3149 (93.2)	3685 (72.2)	−62.2 (−64.5 to −59.8)	<.001
SNP classification					
C-SNP	313 (18.1)	60 (1.8)	373 (7.3)	16.3 (14.5 to 18.2)	<.001
D-SNP	827 (47.9)	57 (1.7)	884 (17.3)	46.2 (43.8 to 48.6)	<.001
I-SNP	51 (3.0)	112 (3.3)	163 (3.2)	−0.4 (−1.4 to 0.6)	.49
Not an SNP	536 (31.0)	3149 (93.2)	3685 (72.2)	−62.2 (−64.5 to −59.8)	<.001
Mean monthly premium, $^c^					
0	1673 (96.9)	2801 (82.9)	4474 (87.6)	14.0 (12.4 to 15.5)	<.001
0.02 to 23.81	18 (1.0)	193 (5.7)	211 (4.1)	−4.7 (−5.6 to −3.8)	<.001
23.82 to 60.25	14 (0.8)	196 (5.8)	210 (4.1)	−5.0 (−5.9 to −4.1)	<.001
60.26 to 261.10	22 (1.3)	188 (5.6)	210 (4.1)	−4.3 (−5.2 to −3.4)	<.001
Authority used to offer benefit					
VBID	721 (41.7)	NA	721 (41.7)	NA	NA
SSBCI	926 (53.6)	NA	926 (53.6)	NA	NA
Both	80 (4.6)	NA	80 (4.6)	NA	NA
Prior authorization					
Yes	299 (17.3)	NA	299 (17.3)	NA	NA
No	1427 (82.7)	NA	1427 (82.7)	NA	NA
Referral required					
Yes	43 (2.5)	NA	43 (2.5)	NA	NA
No	1683 (97.5)	NA	1683 (97.5)	NA	NA

Abbreviations: C-SNP, Chronic Special Needs Plan; D-SNP, Dual Eligible Special Needs Plan; HMO, health maintenance organization; HMO-POS, health maintenance organization–point of service; I-SNP, Institutional Special Needs Plan; NA, not applicable; PFFS, private fee for service; PPO, preferred provider organization; SNP, Special Needs Plan; SSBCI, special supplemental benefits for the chronically ill; VBID, value-based insurance design.

^a^ Plans are described at the contract-plan level. Cells with NA are for variables unique to plans offering the Food and Produce benefit.

^b^ Categories defined by quartiles.

^c^ Categories defined by tertiles among plans with nonzero premiums.

In unadjusted models, a county with a 1-PP higher rate of food insecurity was associated with 2.5-PP (95% CI, 2.1-2.9 PP) higher enrollment in plans offering the Food and Produce benefit (*P* < .001) (Figure, A). Adjusting for plan characteristics did not meaningfully change the association (Figure, B). Additional adjustment for the percentage of plans offering Food and Produce benefits and the percentage that were SNPs attenuated the association by 26% (1.8 PP; 95% CI, 1.3-2.2 PP; *P* < .001) (Figure, C). Further adjustment for the percentage of MA beneficiaries enrolled in SNPs attenuated the association by an additional 86% (0.2 PP; 95% CI, 0.0-0.5 PP; *P* = .04) (Figure, D).

**Figure.**
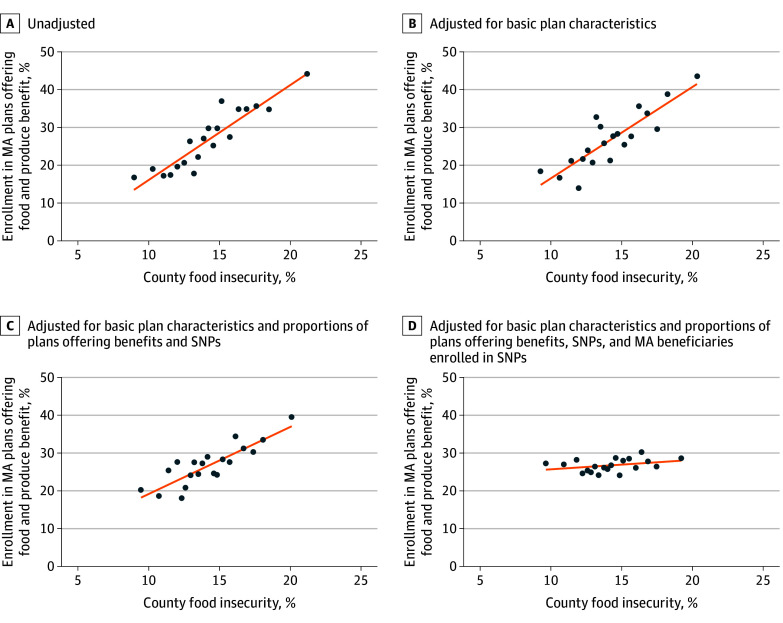
Association Between Enrollment in Medicare Advantage (MA) Plans Offering Food and Produce Supplemental Benefits and County-Level Food Insecurity Dots reflect mean percentage enrollment in MA plans offering the Food and Produce benefit across counties by ventile of county-level food insecurity, as measured by the 2025 Feeding America Map the Meal Gap^6^ project and weighted by county-level MA enrollment. B, Adjusted for basic county-level plan characteristics, including the number of MA plans and MA penetration (the proportion of Medicare beneficiaries enrolled in MA) as well as mean star ratings (in categories) and mean premiums (in quartiles) weighted by enrollment in each plan. C, Additionally adjusted for the proportion of MA plans offering the Food and Produce benefit and the proportion that were Special Needs Plans (SNPs). D, Additionally adjusted for the proportion of MA beneficiaries enrolled in SNPs.

## Discussion

We found that SNP enrollment—rather than plan availability—largely explained the association between county-level food insecurity and enrollment in plans offering Food and Produce benefits. SNPs were more likely to offer such benefits, and individuals in counties with greater food insecurity were more likely to select these plans. The descriptive nature of our study precludes assessment of causality, and limited data availability prevents evaluation of benefit utilization.

Our study found little evidence that plans were preferentially offering Food and Produce benefits in more food insecure counties. Improved targeting of these benefits could better support communities most in need.

## Notes

Supplement 1.
eMethods.

eReferences.

Supplement 2.
Data Sharing Statement
