# Supplementary material for: County-Level Food Insecurity and Access to Medicare Advantage Food Benefits
Source: JAMA Netw Open. 2025 Dec 10;8(12):e2548223. doi: 10.1001/jamanetworkopen.2025.48223 (PMC12696596; doi:10.1001/jamanetworkopen.2025.48223)
Supplement: Supplement 1. — eMethods. eReferences. [file jamanetwopen-e2548223-s001.pdf]

Supplemental Online Content

Kumar M, Bond AM, Khullar D, Schpero WL. County-level food insecurity and access to Medicare Advantage food benefits. *JAMA Netw Open*. 2025;8(12):e2548223. doi:10.1001/jamanetworkopen.2025.48223

eMethods.

eReferences.

This supplemental material has been provided by the authors to give readers additional information about their work.

© 2025 Kumar M et al. *JAMA Network Open*.

eMethods

Details on Data Sources

1. Medicare Advantage (MA) Data (Centers for Medicare & Medicaid Services)[1]
    a. Medicare Advantage Landscape File (2025)
        i. Data: MA Plan Characteristics Data
    b. Monthly Enrollment by Contract, Plan, State, County (CPSC)
        i. Contract Information (Feb 2025)
            1. Data: Contract Effective Date
        ii. Enrollment Information (Feb 2025)
            1. Data: Plan County Enrollment
    c. Plan Benefits Package (2025)
        i. Data: Food and Produce Benefits data.
    d. MA State County Penetration (Feb 2025, July 2024 for Connecticut (CT))
        i. Data: County MA enrollment
2. Food Insecurity Data (Feeding America Map the Meal Gap)[2]
    a. 2025 report (2023 data) for most counties
    b. 2024 report (2022 data) for Kalawao County, HI (FIPS: 15005)
    c. 2023 report (2021 data) for CT counties

Details on Methods:

The following plan types available in the Medicare Advantage Landscape File were excluded from analysis: Cost, Medicare-Medicaid Plans (MMP), Demonstrations, stand-alone Prescription Drug Plans (PDP), Employer sponsored (Plan ID ≥ 800), Part B only, and National Program of All Inclusive Care for the Elderly (PACE).

CMS suppresses data on MA plan enrollment within a county when enrollment is 10 or less. These observations (which represented 0.84% of total MA enrollment) were imputed with a value of 5. In 11 counties where this imputation resulted in a percent enrolled in plans with the Food and Produce benefit > 100, the imputed enrollment value was county MA enrollment divided equally among all plans in the county. A sensitivity analysis revealed that results were not meaningfully affected by imputation. CMS also suppresses total county MA enrollment when values are less than 10. Given lack of data, 7 counties with no county MA enrollment data were excluded from this analysis.

Plans were defined at the contract number-plan ID level; we leveraged segment IDs to identify county-level variation in plan benefits.

The 2025 Feeding America Map the Meal Gap dataset represents the most recent year for which complete county-level food insecurity data are available. This dataset did not include information for Kalawao County, HI (FIPS: 15005), which we abstracted from the 2024 version of the dataset. Researchers from Feeding America estimate county-level food insecurity rates using a two-step model. First, they build a regression model using state-level data from the *2009-2023 Current Population Survey: Food Security Supplement* to identify how variables like unemployment, poverty, income, race/ethnicity, homeownership, and disability predict food insecurity. Then, they apply the resulting coefficients for these variables to corresponding county-level data from the American Community Survey and Bureau of Labor Statistics to generate predicted county-level food insecurity rates[3]. Food insecurity data from the Map the

© 2025 Kumar M et al. *JAMA Network Open*.

Meal Gap Project have been widely used in literature to examine relationships between food insecurity and health[4-8].

For Connecticut only: CT recently transitioned from 8 counties to 9 county-equivalent “planning regions.” We relied on 2024 MA enrollment data for CT counties given the more recent versions of this data source do not contain CT data due to this transition. The 2023 Map the Meal Gap report was used for CT as this was the last year Feeding America reported CT data at the county level.

© 2025 Kumar M et al. *JAMA Network Open*.

References

1. Data from: Medicare Advantage/Part D Contract and Enrollment Data. 2025. Centers for Medicare and Medicaid Services
2. Data from: Map the Meal Gap. 2023-2025. Feeding America
3. Dewey AH, Julie; Dawes, Sena; Harris, Virginia; Hake, Monica; Engelhard, Emily. *Map the Meal Gap: A Report of Local Food Insecurity and Food Costs in the United States in 2023*. 2025. https://map.feedingamerica.org/
4. Song S, Trisolini MG, LaBresh KA, Smith SC, Jr, Jin Y, Zheng Z-J. Factors Associated With County-Level Variation in Premature Mortality Due to Noncommunicable Chronic Disease in the United States, 1999-2017. *JAMA Network Open*. 2020;3(2):e200241-e200241. doi:10.1001/jamanetworkopen.2020.0241
5. Li Q, Zhao S, Çakır M, Yu Z. Association of Supplemental Nutrition Assistance Program Retailers With Child Food Insecurity During the COVID-19 Pandemic. *JAMA Pediatrics*. 2023;177(4):430-431. doi:10.1001/jamapediatrics.2022.5984
6. Vieira de Oliveira Salerno PR, Cotton A, Elgudin YE, et al. Social and Environmental Determinants of Health and Cardio-Kidney-Metabolic Syndrome–Related Mortality. *JAMA Network Open*. 2024;7(9):e2435783-e2435783. doi:10.1001/jamanetworkopen.2024.35783
7. Berkowitz SA, Basu S, Gundersen C, Seligman HK. State-Level and County-Level Estimates of Health Care Costs Associated with Food Insecurity. *Prev Chronic Dis*. Jul 11 2019;16:E90. doi:10.5888/pcd16.180549
8. Beldon MA, Clay SL, Hughes MC, Mazurek K. The Relationship Between Food Insecurity and Low Birthweight Across US Counties and Related Racial Disparities. *J Racial Ethn Health Disparities*. Feb 26 2025;doi:10.1007/s40615-025-02325-9

© 2025 Kumar M et al. *JAMA Network Open*.
