# Supplementary material for: County-Level Food Insecurity and Access to Medicare Advantage Food Benefits
Source: JAMA Netw Open. 2025 Dec 10;8(12):e2548223. doi: 10.1001/jamanetworkopen.2025.48223 (PMC12696596; doi:10.1001/jamanetworkopen.2025.48223)
Supplement: Supplement 2. — Data Sharing Statement [file jamanetwopen-e2548223-s002.pdf]

Data Sharing Statement

Kumar. County-Level Food Insecurity and Access to Medicare Advantage Food Benefits. *JAMA Netw Open*. Published December 10, 2025. doi:10.1001/jamanetworkopen.2025.48223

Data
Data available: Yes
Data types: Data (not involving human participants)
How to access data: All data used in this study are publicly available from the Centers for Medicare and Medicaid Services (https://www.cms.gov/data-research/statistics-trends-and-reports/medicare-advantagepart-d-contract-and-enrollment-data) and Feeding America (https://map.feedingamerica.org/).
When available: With publication

Supporting Documents
Document types: Statistical/analytic code
How to access documents: The statistical/analytic code for this analysis will be made available upon request to the first author (mak4037@med.cornell.edu).
When available: With publication

Additional Information
Who can access the data: Data will be available to anyone requesting the data.
Types of analyses: Data will be made available for any purpose.
Mechanisms of data availability: Data will be made available to anyone requesting the data.
